# Divergent sensory transcriptomic profiles in positive and negative learning in *Bicyclus Anynana* butterflies

**DOI:** 10.1007/s00359-025-01771-4

**Published:** 2025-10-22

**Authors:** Yi Ting Ter, Erica L. Westerman

**Affiliations:** 1 Department of Biological Sciences , University of Arkansas , Fayetteville, AR 72701 USA; 2 Department of Genetics and Biochemistry , Clemson University , Clemson, SC 29634 USA

**Keywords:** Valence, Visual learning, Mate choice, Social learning, Neurogenomics, Lepidoptera

## Abstract

Mate preference learning, where individuals learn to prefer or avoid specific phenotypes during mate selection, is pervasive across animal taxa and influences reproductive isolation and trait evolution. Despite its significance, the genetic basis underlying the associated valence attribution (whether to prefer or avoid) remains largely unclear. Both in terms of what genes are associated with attributing valence, and whether valence attribution is associated with transcriptional changes in both sensory tissues and neural circuits in the brain, or is restricted to the brain. Here, we investigate the neurogenomic basis of positive and negative mate preference learning using *Bicyclus anynana* butterflies. We put females in positive or negative training scenarios, as well as a naïve control, and compared their transcriptomic profiles across three tissues: antennae, eyes, and brain using RNA-sequencing. Our results reveal tissue-specific transcriptional responses, with the antennae showing higher transcriptomic changes during negative training and the eyes showing higher transcriptomic changes during positive training, relative to the naïve control, indicating that valence attribution during learning may not be restricted to transcriptional changes in the brain. We identified a subset of genes in each tissue whose expression patterns changed with valence, as well as genes previously linked to classical conditioning pathways, supporting the hypothesis that imprinting-like learning and classical conditioning share molecular mechanisms. Our findings suggest that valence attribution in mate preference learning involves tissue-specific transcriptional responses in sensory and brain tissues, emphasising the role of peripheral sensory systems in modulating learned mate preferences.

**Supplementary Information:**

The online version contains supplementary material available at 10.1007/s00359-025-01771-4.

## Introduction

Learning is a common process involving the development of behavior, and can occur throughout life. Males and females from many species can learn their mate preferences, and when this experience occurs at an early stage of life (prior to sexual maturity), affecting later mate choice, it is called imprinting (Immelmann 1975; Verzijden and ten Cate 2007; Verzijden et al. 2012). Imprinting has been shown in vertebrates (Lorenz 1937; Cate and Vos 1999; Irwin and Price 1999; Verzijden and ten Cate 2007; Verzijden et al. 2012), famously in birds, as well as in invertebrates (i.e. imprinting-like learning) (Hebets 2003; Westerman et al. 2012; Westerman and Monteiro 2013). Most experimental evidence of imprinting-like learning in insects comes from early exposure of sexually immature adults to the phenotypes of surrounding adult individuals, thereby learning mating preferences (Hebets 2003; Westerman et al. 2012; Westerman and Monteiro 2013). Experiences that induce these learned preferences can be either positive or negative, resulting in a preference or aversion for an observed trait, and facilitating evolutionary consequences such as reproductive isolation between populations, rapid trait evolution, or hybrid swarms (Verzijden et al. 2005; Verzijden and ten Cate 2007). The pervasiveness and evolutionary impact of mate preference learning make understanding the genetic and neurobiological mechanisms underlying this behavior important.

One critical element of preference learning is valence attribution, the process of assigning positive or negative value to stimuli. To date, valence attribution has been primarily studied in learning contexts other than mate preference, and in higher-order brain regions rather than sensory organs. In vertebrates, brain structures such as the amygdala, nucleus accumbens, and prefrontal cortex have been implicated in attributing positive and negative valence to stimuli, integrating sensory information and internal states to guide decision making and responses (reward and aversion) (Bouret and Richmond 2010; Hu 2016; Pignatelli and Beyeler 2019; Nejati et al. 2021). Similarly, in invertebrates, the mushroom body plays a key role in encoding learned valence (Aso et al. 2014; Eschbach et al. 2021; Noyes and Davis 2023). While this brain region is similarly likely to be involved in assigning mate preference learning valence, sensory structures also have the potential to play a key role. Sensory receptor neurons are the first to interact with cues, such as visual signals or pheromones, and recent evidence suggests that peripheral systems may not merely relay information but actively contribute to valence coding (Wu et al. 2022). Understanding if, and how, sensory systems can play a role in valence attribution could provide a more comprehensive view of how mate preference learning works.

Imprinting-like learning, a form of mate preference learning, is often thought to be neurobiologically distinct from associative learning. This belief largely stems from the observation that mate preferences formed during an early, sensitive developmental period are often hard to modify by later experiences (Immelmann 1975; Cate and Vos 1999; Irwin and Price 1999). However, this distinction remains a subject of debate. Some researchers propose that imprinting learning may be based on molecular mechanisms similar to those found in classical conditioning. In this view, a trainer individual (sexually mature adult) can be seen with a combination of many stimuli, some of which could function as an unconditioned stimulus (US), such as movement, and become associated with a conditioned stimulus (CS), like a potential mate’s color or odor (Eiserer 1980; Bolhuis et al. 1990). While the genetic and neurobiological mechanisms involved in positive and negative classical conditioning (a form of associative learning) have been extensively studied (Dudai et al. 1976; Byers et al. 1981; Tully and Quinn 1985; Levin et al. 1992; Swank and Bernstein 1994; Mokin and Keifer 2005; Unoki et al. 2005; Mizunami et al. 2009), the processes underlying positive and negative imprinting-like learning remain poorly understood. As a result, it remains challenging to compare the neural and genetic bases of associative and imprinting-like learning.

Although classical conditioning and valence attribution have been studied in model insects like *Drosophila melanogaster* (Dudai 1977; Tully and Quinn 1985; Aso et al. 2014; Eschbach et al. 2021; Noyes and Davis 2023), studies in Lepidoptera remain limited. Some work in moths and butterflies have shown associative learning capabilities in response to olfactory and visual cues (Fan et al. 1997; Satoh et al. 2016; Young et al. 2024), yet the molecular basis of valence-specific learning, such as the distinction between positive and negative associations, remains poorly understood in this group, especially in the context of imprinting-like learning. Here, we utilize a species that exhibits both positive and negative imprinting-like learning, the butterfly *Bicyclus anynana*, to identify neurogenomic differences associated with mate preference learning valence. In this species, females can develop preferences for novel male traits (number of dorsal forewing eyespots) when paired with an undisrupted sex pheromone blend (positive training) (Westerman et al. 2012), or learn to avoid the wild-type spot phenotype when paired with a disrupted sex pheromone blend (negative training) (Westerman and Monteiro 2013). These learned mate preferences influence mate choice decisions, contributing to preference or aversion to the spot phenotype. This study aims to uncover the molecular mechanisms underlying mate preference learning in *B. anynana*. Specifically, we aim to elucidate genes encoding for positive and negative valence in mate preference learning, to what extent sensory (eye and antennae) and brain tissues drive transcriptomic changes, and the conserved neural circuitry between mate preference learning and other learning processes.

Along these lines, we tested four hypotheses related to mate preference learning. First, we tested whether changes in attributed valence (positive vs. negative association with a visual signal) are primarily driven by transcriptional changes in the brain rather than sensory tissues (eyes and antennae). Second, we tested whether the sensory tissues processing the unconditioned stimulus (olfaction; antennae) and conditioned stimulus (vision; eyes) respond similarly to specific learning contexts, for instance, a greater transcriptomic response during negative training over positive training due to negative training exhibiting a stronger behavioral response than positive training (Roussel et al. 2012; van der Schaaf et al. 2022). Third, we tested the hypothesis that molecular processes underlying learning are conserved across valence scenario, identifying genes that may regulate general learning processes across contexts (e.g. upregulated in both contexts) and others that may encode for signal valence through context-dependent regulation (e.g. upregulated in one, but downregulated in another). Lastly, we tested whether imprinting-like learning shares molecular similarities with classical conditioning, focusing on genes involved in immediate early responses, neurotransmitter signalling, and synaptic plasticity.

## Materials and methods

### Study species and animal husbandry

*Bicyclus anynana* is a sub-Saharan, African butterfly that has been reared in the lab since 1988 (Brakefield and Reitsma 1991). The colony at the University of Arkansas was established in 2017, with ~ 1,000 eggs from a colony at the National University of Singapore. *B. anynana* colonies at the University of Arkansas are reared in a climate controlled, USDA-APHIS approved (Permit # P526P-20–00417) greenhouse. The greenhouse was maintained at 27 °C, with a relative humidity of 60–80%, natural lighting augmented by Sun Blaze T5 high output 120 V fluorescent light fixtures (including UV wavelengths) and a 13:11 h light: dark photoperiod. The larvae were fed with maize plants (*Zea mays*) (Jollytime popcorn) *ad libitum* and adults were fed with bananas. Adults that emerged on the same day (Day 0) were transferred to sex and age-specific cages (32 × 32 × 32 cm; Bioquip), ensuring that all butterflies used in this study were virgins prior to being used in exposure assays. Since the laboratory colonies are large (~ 1000), there is sufficient genetic diversity found in our colonies, and it is comparable to the genetic diversity found in natural populations (Beldade et al. 2006; De Jong et al. 2013).

### Transcriptomic analysis of female brain, eyes and antennae tissues

#### Training assays and sample collection

**Fig. 1:**
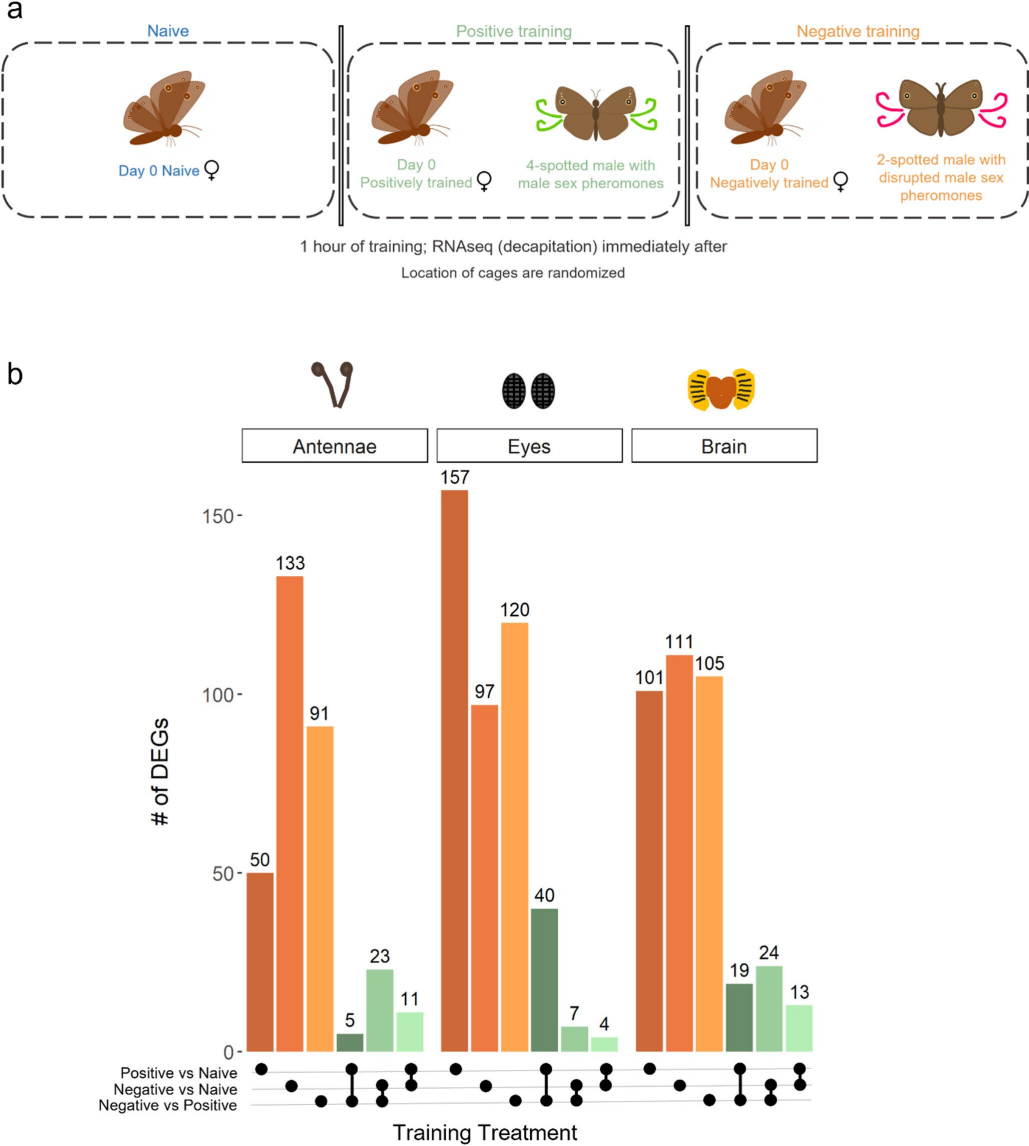
Experimental setup of training assays (**a**) and number of DEGs in different comparisons of interest in antennae, eyes and brain tissues. **a** Each female and male were size- and age-matched (Day 0 for females and Day 3 for males). Males were marked with UV paint and nail polish on Day 2, one day prior to training, and placed into their respective training cages to allow for acclimation. All training assays were conducted in rectangular cages measuring 40 × 40 × 60 cm, with white opaque blinders between each cage to prevent visual cues between treatments. In the control treatment, newly emerged, naïve females were isolated in a cage for 1 h. In the positive training treatment, newly emerged females were exposed for 1 h to a 4-spotted male with intact sex pheromones. In the negative training treatment, newly emerged females were exposed for 1 h to a 2-spotted male with disrupted sex pheromones. Immediately following the 1 h training period, all females were decapitated and flash frozen for RNAseq. **b** Broadly speaking, orange bar charts indicate uniquely DEGs (i.e. only in one social training context) and green bar charts indicate shared DEGs (i.e. in two social training contexts). Black dots indicate the comparison of interest (e.g. Positive vs. Naive), and lines connecting two dots indicate DEGs shared between comparisons

To determine the gene expression associated with different types of social learning, we exposed female *B. anynana* to different training/exposure treatments for one hour (*n* = 10 per treatment) from August to October 2022. Exposure assays were conducted one hour after sunrise, in a rectangular mesh cage (40 × 40 × 60 cm; Bioquip) under our greenhouse conditions as described above. Every individual used in our exposure assays was size- and age-matched: females were Day 0, ensuring sexual immaturity (Costanzo and Monteiro 2007), and males were Day 3, when their sex pheromones are known to be attractive to females (Nieberding et al. 2012). Naïve females were isolated and were not exposed to training stimuli (i.e. males), females that were exposed to a randomly selected 4-spotted male with undisrupted male sex pheromones were considered given a “positive training exposure”, and females that were exposed to a randomly selected 2-spotted male with disrupted male sex pheromones were considered given a “negative training exposure”, based on prior studies (Westerman et al. 2012; Westerman and Monteiro 2013) (Fig. 1a). We acknowledge that these training treatments have both different visual and different olfactory stimuli, inducing potential stimuli confounding factors. However, it is the pairings of these two sets of stimuli (4-spots plus undisrupted pheromone and 2-spots plus disrupted pheromone) that produce a positive learning outcome (learn to prefer) and a negative learning outcome (learn to avoid) respectively in our system.

Both naïve and trained females were placed in identical individual cages, and experienced the same environmental conditions; the only difference between them was the presence/absence of a male during the exposure period. Individual cages were separated by white corrugated plastic sheets, so females could not see butterflies or training scenarios outside of their own cage (Fig. 1a). Treatments were conducted concurrently to control for stochastic random effects of greenhouse conditions, and the physical location of cages for different treatments (naïve, positive training exposure, and negative training exposure) were rotated to account for any possible random effects of cage location.

To create the 4-spotted male phenotype for the positive training exposure assays, we painted two extra spots using UV-reflective white paint (Reel Wings; North Dakota, USA) on the dorsal forewing, in between the two natural eyespots, as previously described (Westerman et al. 2012). To create the 2-spotted male phenotype, we painted two spots directly on the natural dorsal forewing eyespots. Thus, male butterflies had different numbers of dorsal forewing UV-reflective eyespots (2- vs. 4-spots), but the same amount of UV reflective paint. To disrupt male sex pheromones for the negative training exposure assays, we painted Revlon Liquid Quick Dry nail polish (Revlon; New York, USA) over the androconia on the ventral fore- and hindwings of males, as described in (Westerman and Monteiro 2013). All male manipulations were done a day before behavioral/exposure assays, and males were placed into rectangular cages for 24 h prior to use in assays to allow for recovery from wing manipulation (Westerman et al. 2012; Westerman and Monteiro 2013). We determined whether matings occurred during the training assay by dusting the ends of females’ abdomens with fluorescent clownfish orange powder (Risk Reactor Inc; California, USA), and checking the abdomen ends of males using a blacklight flashlight. If a female had mated with a male during training, they would not be used for subsequent analysis due to the possible effect of mating on brain gene expression. However, in our training exposure assays, no matings occurred, so none of our exposed females were excluded.

#### RNA extraction and cDNA library Preparation

After one hour of exposure (training), the females were decapitated with RNA-free scissors. Each head was placed into individual RNA-free 1.5 ml LoBind tubes, immediately flash frozen in liquid nitrogen, and stored in −80 °C until dissection. To prevent RNA degradation during dissection, we soaked the heads in 500 ul of prechilled RNAlater ICE (Ambion; Texas, USA) and kept them in −20 °C at least 16 h before dissection and RNA extraction. We then dissected the heads in RNAlater ICE and extracted RNA using the Nucleospin miRNA kit (Macherey-Nagel; Duren, Germany), which extracts both large RNA (> 200 bp) and small RNA (< 200 bp). We utilized large RNA for downstream library preparation and analyses. Quality of RNA were determined using Nanodrop 2000 (Thermo Fisher Scientific; Massachusetts, USA). We prepared RNA libraries using the KAPA mRNA HyperPrep Kit and Unique Dual-Indexed Adaptors (KAPA Biosystems; Massachusetts, USA), with 200 ng of RNA as input, and shipped to the University of Chicago’s Genomics Facility on dry ice. The libraries were further accessed for quality using a 5300 Fragment Analyser (Agilent; California, USA), followed by a 50 bp paired-end sequencing across four lanes of NovaSeq-X-Plus (Illumina; California, USA).

Since RNAseq is a destructive process, and we were interested in what was going on in the brains and sensory organs of individuals while they were being trained, we could not assess whether each female had successfully learned via mate choice assays two days after the training exposure (and associated decapitation) took place. However, based on prior work showing that 70% of exposed females typically establish learned preferences (Westerman et al. 2012; Westerman and Monteiro 2013), we used a sample size of 10 per treatment to help mitigate the impact of any individuals in our collection that did not learn. While a sample size of 10 is larger than is usually used for RNASeq studies, this sample size is based on the known variance in preference learning in our system, and ensures that a majority of individuals from the positive and negative training treatments would have learned. While this approach may mask some subtle expression differences that might be important for the learning process, it identifies genes of larger effect that are associated with the imprinting-like learning process.

#### Differential gene expression analysis

We concatenated the raw fastqc files from each library and accessed their quality using FASTQC v0.11.5, removed adaptor sequences using Trimmomatic v0.36, and aligned the trimmed sequences to *B. anynana* reference genome (v1.2) (Nowell et al. 2017) using STAR v2.7.10a, instead of *B. anynana* genome (v1.1) (Saccheri 2023) as the two genomes are derived from butterflies from different *B. anynana* populations, and our *B. anynana* population has a 10% better alignment to the Nowell et al. (2017) reference genome than it does to the Saccheri (2023) reference genome (Fig. S1).

The read counts generated by STAR were used as input for differential expression analyses using the “DESeq2” package (Love et al. 2014). We created tissue-specific datasets and used the GLM “y ~ train”, where train represented the different social exposure treatments (e.g. positive, negative and naive) in different tissues. Genes with a total read alignment count of < 10, and outliers were filtered out and not included in the differential expression analysis. Gene expression was calculated as the log of the expression fold change (log_2_FC) and the “apeglm” method (Zhu et al. 2019) was used for log_2_FC shrinkage. We then calculated empirical FDR for each gene by performing permutation tests by generating 1000 permutated datasets by randomly assigning each sample name for each tissue’s dataset in R (Ghalambor et al. 2015; Bloch et al. 2018) (*N* = 30 per tissue). We then performed GLM in the same way as the original DESeq2 analyses, generating a null distribution of 1000 permutated p-values per gene transcript, and a gene was considered differentially expressed when its associated p-value fell below the 1% tail of the permutated data p-value distribution (i.e. empirical threshold). This method has been shown to better capture the structure of data and does not assume gene independence (Slonim 2002). Therefore, this reduces the risk of overcorrection compared to other multiple test correction methods, since gene expression is likely influenced by other associated genes, and thus not independent. Pairwise comparisons of interest within each tissue (i.e. Positive vs. Naive, Negative vs. Naive, Negative vs. Positive; Treatment on the right is the reference level) were extracted and analyzed using the “DESeq2” package (Love et al. 2014).

RNA-sequencing generated a total of approximately five billion high quality 50 bp, paired- end (PE) reads (Table S1). Adapter trimming removed an average of 0.4 million reads per sample (1%) and approximately 87.5% of the remaining trimmed reads mapped to the *B. anynana* genome v1.2. Across the tissue libraries, antennae had 17,908 genes with at least 10 mapped reads (79% of 22,642 annotated genes), brain had 17,624 genes with at least 10 mapped reads (78% of 22,642 annotated genes), and eyes had 17,043 genes with at least 10 mapped reads (75% of 22,642 annotated genes) which were used for differential expression analyses.

#### Getting orthologs and categorizing them into gene groups

We then used BLASTP and BLASTX in DIAMOND v2.1.7 (Buchfink et al. 2015, 2021) to align the protein and cDNA sequences in our reference genome to the NCBI database respectively. The top candidate ortholog was determined based on the lowest E-value. In cases where numerous hits had identical E-values, we selected based on the highest hit score. If there were no hits using either BLASTP or BLASTN, the gene was defined as NA. We then further blasted our reference genome to the *D. melanogaster*’s genome, to get *D. melanogaster* orthologs. For any gene that did not have a *D. melanogaster* ortholog, we manually queried it into FlyBase (Gramates et al. 2022) using their “Homolog” QuickSearch tool, allowing us to find *D. melanogaster’s* orthologs in other model organism genes. Gene ontology (GO) terms for all differentially expressed genes were annotated using Blast2GO (Götz et al. 2008), Flybase (Gramates et al. 2022), Ernst and Westerman (2021).

### Classifying categories of differentially expressed genes (DEGs)

#### Identifying DEGs associated with Valence of signal

To identify genes putatively associated with assigning valence, we set the range for expression in Naïve females to be between − 0.075 and 0.075, and set the positive and negative log2FC values as either (1) Pos < −0. 5 and Neg > 0. 5, or (2) Neg < −0.5 and Pos > 0.5. This extracted a subset of DEGs that had no/low differential expression in the Naïve treatment, but high and antagonistic differential expression between Pos and Neg treatments. We then cross-referenced this list to the Neg-Pos DEG list.

#### Identifying DEGs that are associated with classical conditioning

To identify genes putatively associated with both classical conditioning and imprinting-like learning, we first focused on DEGs that were grouped into “Learning and Memory”, “Neurotransmitter Receptors” and “Nervous System”. These DEGs were then cross-referenced to other research that specifically mentioned using a classical conditioning paradigm, i.e. forming an association between a US and CS (Eelen 2018), ranging across different taxa such as invertebrates (fruitflies, crickets) and vertebrates (mice, rats, zebra finches, chicks).

### Ethical statement

All *B. anynana* butterflies were maintained in laboratory conditions specified by U.S. Department of Agriculture APHIS permit (# P526P-20–00417). Butterflies were fed with *ad libitum* food and water. Those used in this experiment were decapitated quickly and flash frozen immediately after for this and future studies.

## Results

### Sensory organs and brain exhibit different transcriptomic profiles across valence-based training conditions

To test our hypothesis that changes in attributed valence are primarily driven by transcriptional changes in the brain rather than sensory organs, we identified differentially expressed genes (DEGs) between females exposed to positive and negative training conditions (positive vs. negative). It is important to note that because the positive and negative training conditions involve distinct sensory cues (e.g., different pheromone blends and spot numbers), the DEGs identified between these contexts may partly reflect differences in the different sensory stimuli received during training, rather than being specific to the valence learning process. All three tissues examined (antennae, eyes, and brain) exhibited DEGs, which were unique for the different valence training scenarios (Fig. 1b; TableS2–S4). We also observed tissue-specific patterns of gene expression associated with positive and negative mate preference training. Of the three tissues we examined, the antennae, the sensory organ associated with the detection of the pheromone (unconditioned stimulus), exhibited the highest number of DEGs during negative training, and the lowest during positive training; and the eyes, the sensory organ associated with sensing the male wing pattern (conditioned stimulus), exhibited the highest number of DEGs during positive training, and the lowest during negative training. The antennae exhibited 133 DEGs unique to the negative training vs. naïve contrast (Neg), 50 DEGs unique to the positive training vs. naïve contrast (Pos), and 91 DEGs unique between valence contexts (negative training vs. positive training contrast; Neg-Pos; Fig. 1b). The eyes exhibited 97 DEGs unique to Neg, 157 DEGs unique to Pos, and 120 DEGs unique between valence contexts Neg-Pos (Fig. 1b). The brain exhibited 111 DEGs unique to Neg, 101 DEGs unique to Pos, and 105 DEGs unique between Neg-Pos (Fig. 1b). We also found differential expression of sensory genes, including olfactory receptors (*Or92a*) and vision genes (e.g., *Patj*, *rst*,* ogre*,* Optix*) supporting the sensory systems’ involvement in valence processing (Fig. S4).

### A small set of genes are similarly differentially expressed in both Valence contexts and May be associated with the act of learning independent of Valence

**Fig. 2:**
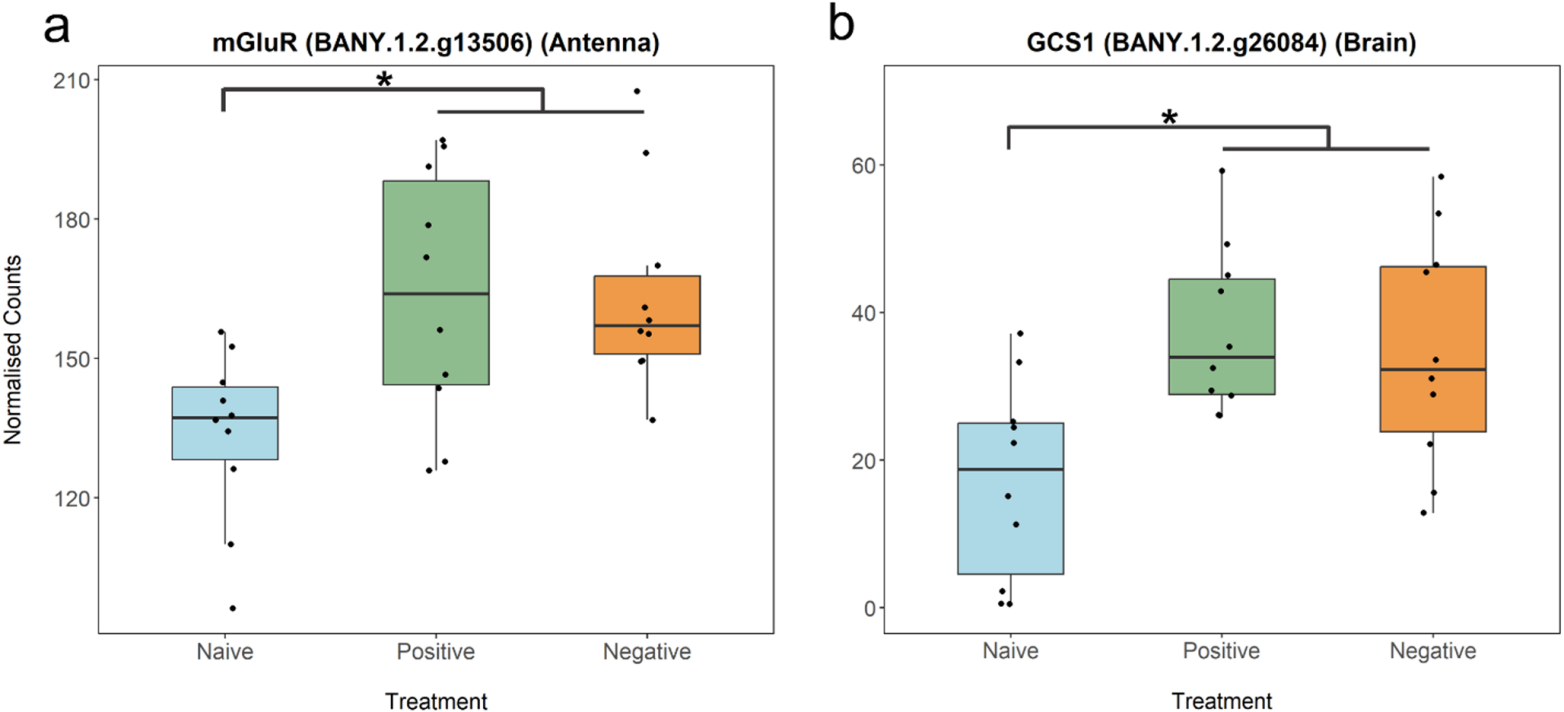
Box plots of two putative learning genes. **a** metabotropic glutamate receptor (*mGluR*) was upregulated in Pos and Neg contexts compared to Naïve in the antennae. **b** mannosyl-oligosaccharide glucosidase (*GCS1*) was upregulated in Pos and Neg contexts compared to Naïve in the brain. Asterisks indicate significance at/below the 1% permutation threshold

**Fig. 3:**
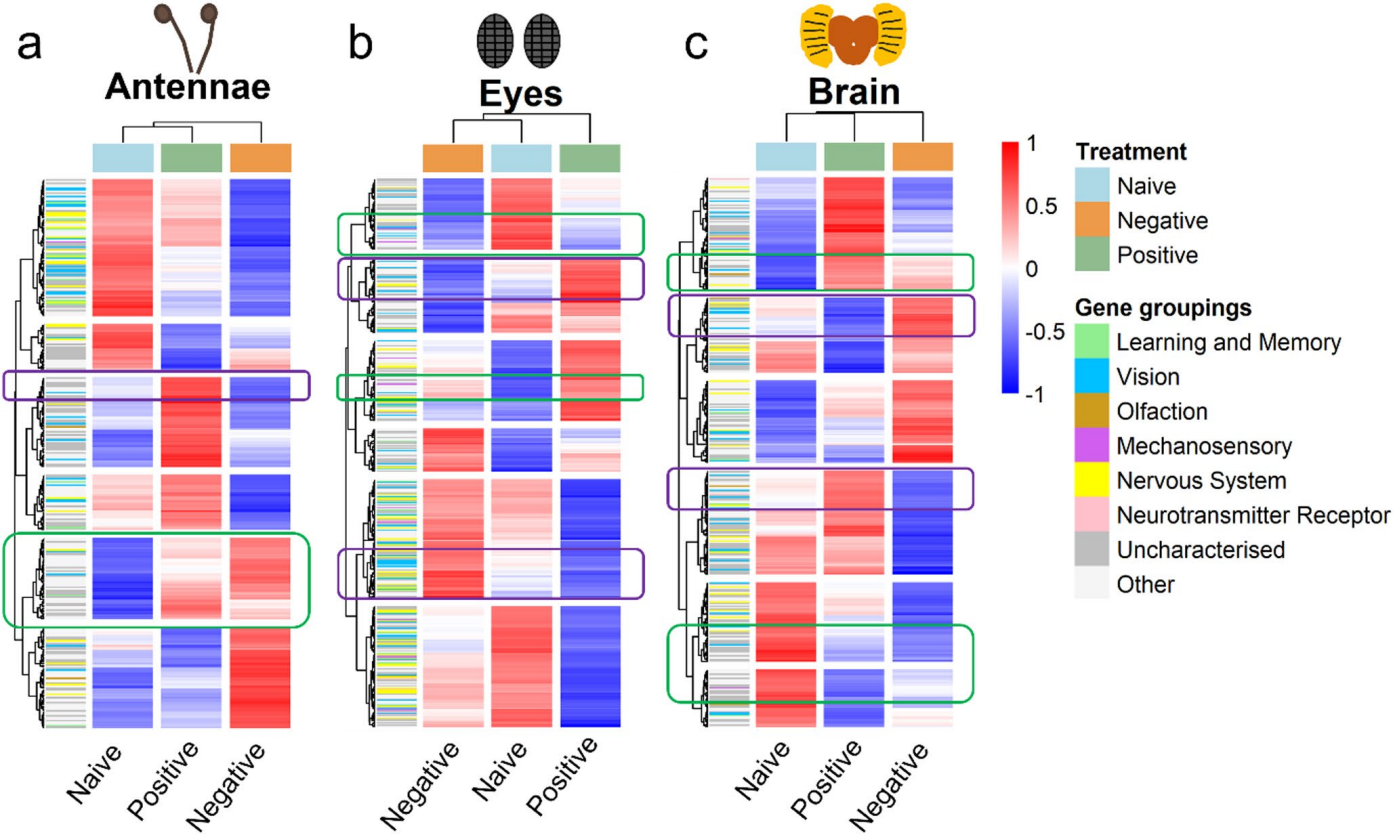
Gene expression heatmaps of DEGs in antennae (**a**), eyes (**b**) and brain (**c**) tissues. Each column indicates a training treatment, and each row indicates a single DEG. Counts were normalised by variance stabilising transformation, and Z-scores were calculated for visualisation and plotting. Genes and training treatment are clustered by expression, with blue colors denoting downregulation of expression and red colors denoting upregulation of expression. Each DEG was further categorized into a gene group, based on putative function. Green boxes represent examples of putative learning genes (similarly high differential expression in Pos and Neg, compared to Naïve) while purple boxes represent examples of valence-associated genes (high but antagonistic expression in Pos and Neg, compared to low expression in Naïve)

To test the hypothesis that molecular processes underlying learning are conserved across valence scenarios, we searched for genes with similar expression patterns in both positive and negative imprinting-like learning training contexts. Using this criteria, we found a small set of DEGs shared across valence training contexts with similar expression patterns, which may be good candidate genes associated with the act of imprinting-like learning itself, independent of valence. Across Pos and Neg training contexts, there were 7 such DEGs in the antennae, 11 in the brain, and 2 in the eyes (Fig. 1b, Table S5-7). Notably, genes such as metabotropic glutamate receptor 1 (*mGluR*), which was upregulated in both training contexts in the antennae, is known to play a role in neural functions such as short- and long-term memory (Kucharski et al. 2007; Schoenfeld et al. 2013; Nisar et al. 2022) (Fig. 2a). Similarly, mannosyl-oligosaccharide glucosidase (*GCS1*), found upregulated in both training contexts in the antennae and brain, is mostly involved in post-translational processes (Stanley et al. 2022), but has also been linked to learning (Tucholski et al. 2013; Zhang et al. 2024) (Fig. 2b). Separately, we cross-referenced all DEGs in our study to orthologous *D. melanogaster* genes in FlyBase and categorized them into functional groups (including “learning and memory”) based on associated GO terms. We identified 162 putative learning and memory transcripts expressed in our system, of which only a small subset overlapped with our DEGs list– 11 in antennae, 9 in eyes, and 4 in brain (Fig. 3; Tables S2 – S4).

### Opposing expression patterns in shared genes across positive and negative training in sensory and brain tissues

**Fig. 4:**
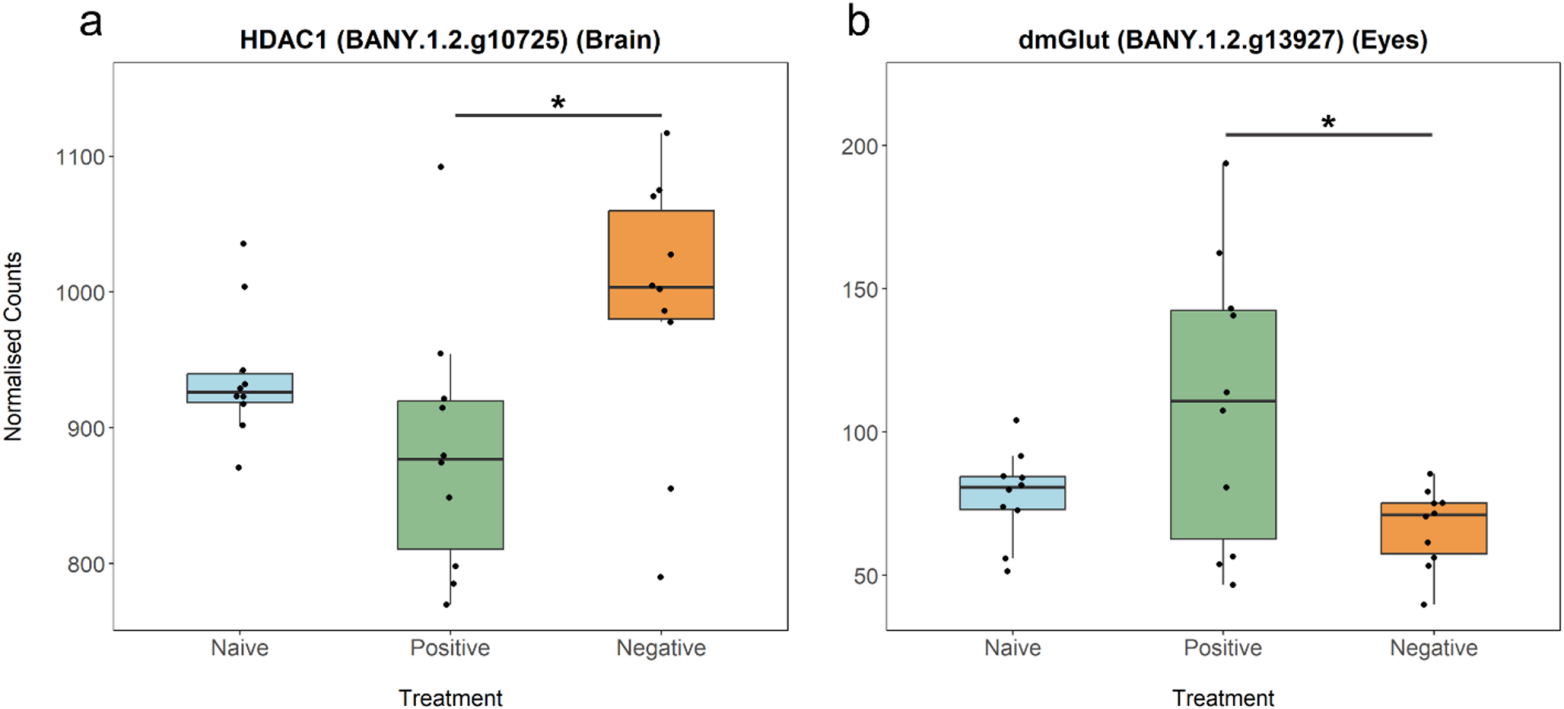
Box plots of putative valence-associated genes. **a** Histone deacetylase 1 (*HDAC1*) was differentially expressed between Pos and Neg; downregulated in Pos and upregulated in Neg. **b** Dietary and metabolic glutamate transporter (*dmGlut*) was differentially expressed between Pos and Neg; upregulated in Pos and downregulated in Neg. Asterisks indicate significance at/below the 1% permutation threshold

To test the hypothesis that some genes encode signal valence through context-dependent regulation, we identified genes exhibiting antagonistic expression patterns across valence contexts. We defined valence-associated genes as genes having a pattern of high, opposing differential gene expression in the Pos and Neg treatments and intermediate expression (relative to the Pos and Neg treatments) in the naïve treatment. Using this criteria, we identified 14 DEGs in the antennae; 32 DEGs in the eyes, and 37 DEGs in the brain (Fig. 3; Table S8-10). Most of these DEGs were either uncharacterised or “Other”. Of particular interest, histone deacetylase (*HDAC1*) was upregulated during negative training exposure, but downregulated during positive training exposure in the brain (Figs. 3c and 4a; Table S9), while dietary and metabolic glutamate transporter (*dmGlut*) was upregulated during positive training and downregulated during negative training in the eyes (Figs. 3b and 4b; Table S10).

### Genes associated with classic conditioning are also associated with imprinting-like learning

**Fig. 5:**
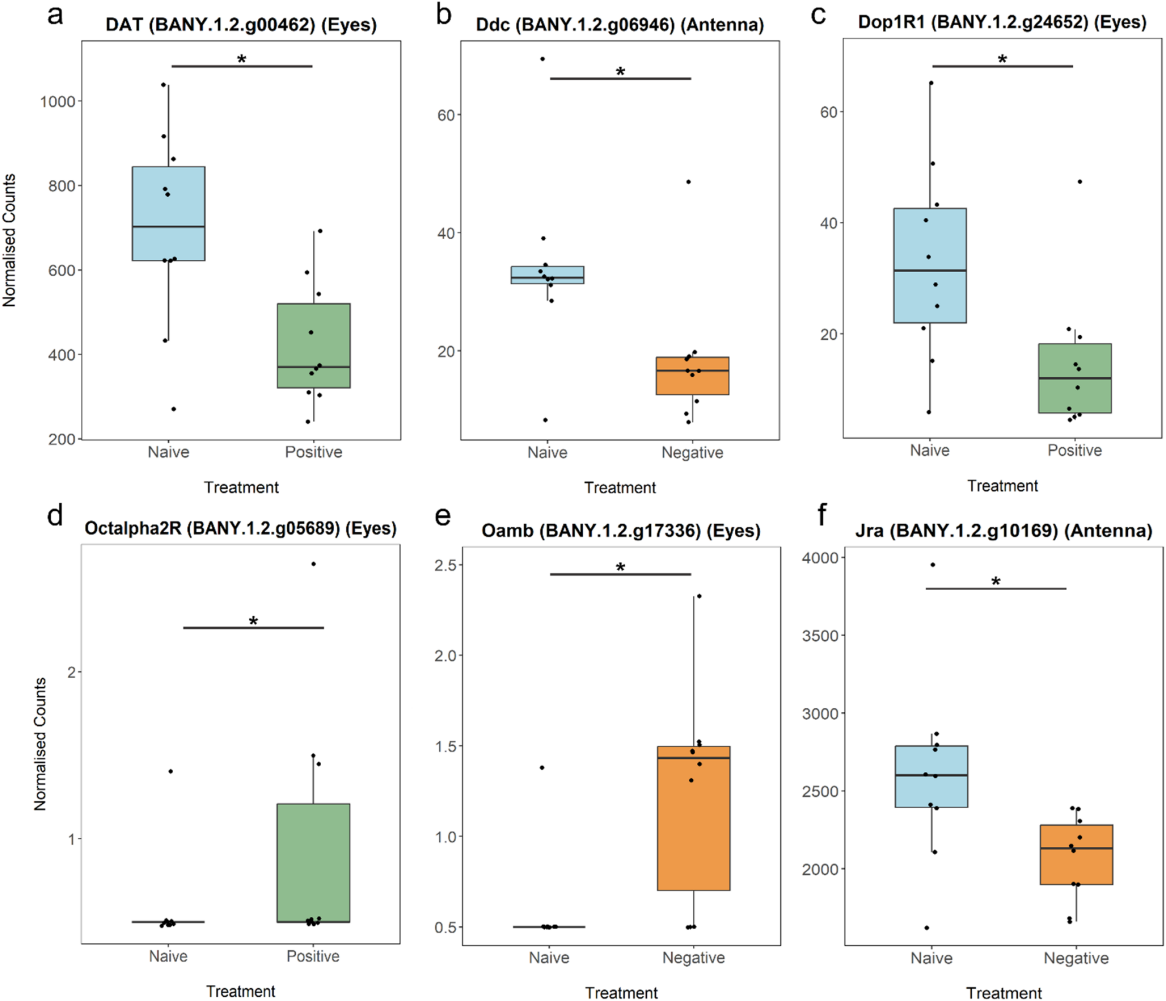
Box plots of genes involved in classical conditioning and imprinting-like learning. **a–c** Differential expression of dopamine transporter (*DAT*), dopa decarboxylase (*ddc*) and dopamine 1-like receptor (*Dop1R1*), that are part of the dopaminergic pathway. **d**,** e** Differential expression of α2-adrenergic‐like octopamine receptor (*Octα2R*) and octopamine receptor 1 (*Oamb*), part of the octopaminergic pathway. **f** Differential expression of an immediate early gene, *Jra/c-jun* in the antennae. Asterisks indicate significance at/below the 1% permutation threshold

Lastly, we tested whether imprinting-like learning shares similar molecular mechanisms with classical conditioning. To narrow down our search for “classical conditioning” genes, we looked at DEGs that are associated with neurotransmitter receptors, learning and memory, and the neuronal system, with a focus on aminergic receptors that have the capacity to modulate neuronal responses. Dopaminergic and octopaminergic signalling has long been implicated in classical conditioning in invertebrates (negative and positive learning respectively). We found dopamine transporters (DAT) and dopamine 1-like receptor 1 (*Dop1R1*) differentially expressed in eyes during Pos training (Fig. 5a–c; Table S4), and octopamine receptor 1 (*Oamb*) and α2-adrenergic‐like octopamine receptor (*Octα2R*) differentially expressed in eyes during Neg and Pos training respectively (Fig. 5d–e; Table S4). Other aminergic receptors, such as acetylcholine and GABA receptors, were also differentially expressed in eyes and antennae during different valence contexts (Fig. S5, Table S4). An immediate early gene (i.e. genes that are transiently expressed and activated quickly in response to a stimulus), *Jra/c-jun*, was upregulated in the antennae during Neg training (Fig. 5f; Table S2).

## Discussion

Valence attribution has traditionally been attributed to higher-order brain regions and neurotransmission (Unoki et al. 2006; Mizunami et al. 2009; Aso et al. 2014; Yamazaki et al. 2018; Eschbach et al. 2021; Noyes and Davis 2023; Xiao et al. 2023), but our results indicate a potential role for sensory organs. Additionally, most differentially expressed genes (DEGs) were unique to each social learning context across tissues, suggesting distinct molecular pathways for encoding positive and negative valence in the brain (Yamazaki et al. 2018; Noyes and Davis 2023). Many chemosensory receptor neurons contribute to valence processing, particularly innate valence, before these signals reach the brain (Knaden et al. 2012). In insects, valence processing has been observed in olfactory sensory neurons (OSNs) that respond selectively to innately attractive or aversive cues. For instance, in *Drosophila melanogaster*, certain OSNs are tuned to detect CO₂, triggering avoidance behavior, while others respond to food-related odors that promote attraction (Suh et al. 2004; Semmelhack and Wang 2009). Furthermore, recent findings demonstrate valence opponency in odorant receptor neurons – such as *Or92a*, which was differentially expressed in our study – suggesting that behavior can be influenced at the peripheral level (Wu et al. 2022). However, most studies focus on innate valence processing in sensory organs, rather than learned (trained) valence. Therefore our findings highlight the importance of peripheral systems in learned valence coding, alongside the brain.

We found distinct, tissue-specific patterns of gene expression associated with positive and negative mate preference learning. The sensory organs detecting the unconditioned stimulus (antennae) exhibited a greater response in the negative preference learning context, while the sensory organs detecting the conditioned stimulus (eyes) exhibited a greater response in the positive preference learning context (Fig. 1b). We initially hypothesized that there would be lower transcriptional response during positive learning and higher transcriptional response during negative training across tissues, as studies have shown that negative training elicits a stronger behavioral response than positive training (Roussel et al. 2012; van der Schaaf et al. 2022). However, the opposing gene expression patterns we found in sensory organs suggests that the differential transcriptomic responses in the antennae and eyes may reflect their respective roles in processing either novel or unpreferred cues. Novel stimuli can trigger increased neuronal plasticity, for instance, in zebrafish, learned sensitivity to new odors during imprinting was accompanied by increased odorant receptor cell neurogenesis (Cui et al. 2017), or in honeybees, where expression of *kakusei*, an immediate early gene, is increased in mushroom bodies and optic lobes during visual learning (Geng et al. 2022). However, it remains unclear whether the observed transcriptomic responses are primarily driven by the novelty of the cue or by the absence of an innate preference for it. It is also possible that the DEGs we observed partly reflect those associated with stimulus detection rather than valence learning, though these cannot be disentangled in our system currently. Future research could address this by identifying neutral cues in this system (i.e. to control for spot number and presence of male), and comparing neurogenomic expression profiles when females are exposed to novel but neutral cues versus novel unpreferred cues. One could also identify other positive and negative mate preference training scenarios to see if these scenarios induce similar sets of DEGs. While the training treatments we used here have both different visual and olfactory stimuli, it is important to note that female *B. anynana* exposed to 2-spotted males with undisrupted male sex pheromones mate preferentially with 2-spotted males in later choice trials (a potential positive training scenario), as do females exposed to 4-spotted males with disrupted male sex pheromones (a potential negative training scenario) (Westerman et al. 2012; Westerman and Monteiro 2013). Since the innate, naïve preference in this system is for 2-spotted males (Robertson and Monteiro 2005; Westerman et al. 2012), it is unclear based on behavioral data whether these two additional training scenarios facilitate learning or are neutral stimuli. But, that could perhaps be explored through future comparative transcriptomic studies. Such studies could disentangle the contributions of sensory stimuli, cue novelty and valence to the observed transcriptomic patterns.

To further explore putative learning genes independent of valence, we identified a small subset of DEGs to be associated with valence-independent learning (Fig. 1b, Table S5-7). Notably, genes such as metabotropic glutamate receptor 1 (*mGluR*) and mannosyl-oligosaccharide glucosidase (*GCS1*) are found upregulated in both training contexts in this study. They have also been linked to learning and memory in other species such as humans and mice (Kucharski et al. 2007; Schoenfeld et al. 2013; Tucholski et al. 2013; Nisar et al. 2022; Zhang et al. 2024). The differential expression across sensory tissues and brain tissues suggests coordinated molecular processes spanning multiple sensory modalities and higher processing tissues. When this subset of genes was cross-referenced with *D. melanogaster* orthologs annotated for learning or memory, we found a small overlap. This limited overlap may indicate that many known learning genes are valence-dependent and expressed only in specific valence contexts. Alternatively, the overlap may be small because our dataset includes genes not yet functionally characterized for learning in other taxa, or that most functional studies to date have focused on other types of learning rather than imprinting-like processes. However, it is important to consider that some of these DEGs might reflect changes in activity levels, or social interactions, rather than learning per se. While trainer male activity levels do not influence whether a female learns to prefer the male’s wing pattern (Table S1 of Westerman et al. 2012), and female activity remains unchanged when exposed to a positively-valenced male in comparison to being isolated in a cage (Ernst et al. 2023) (Table S11), it is unclear whether females alter their activity in response to negatively-valenced males, or how much gene expression is affected simply by the presence of another individual. Nonetheless, we argue that any activity changes in negatively-trained females would still represent a response to the training scenario, which would be mediated by changes in neural activity. Although our findings are correlative, future functional work could test whether these genes causally contribute to learning across taxa, independent of valence context.

To focus more on genes that modulate valence of signal (i.e. the same gene associated with positive and negative valence/learning), we identified valence-associated genes as having a pattern of no/low expression of DEGs in the naive treatment, but high and antagonistic gene expression of DEGs in the positive and negative treatments. In the brain, we found the *HDAC1/Rpd3*, a histone deacetylase, differentially expressed based on valence contexts. There has been growing literature about the involvement of epigenetics during synaptic plasticity and learning (Sultan and Day 2011; Karpova et al. 2017), for instance, changes in acetylation are showed to be involved in learning in mice and sea hares (Bahari-Javan et al. 2012; Mizuno et al. 2012; Mahgoub and Monteggia 2014; McGowan and Roth 2015). However, most studies looking at what molecular mechanisms encode for signal valence do not consider epigenetics, even though epigenetic mechanisms can be within-generation and can be sensitive to the environment and experience (McGowan and Roth 2015; Skinner 2023), leading to valence-dependent plastic gene regulation. It would be interesting to look at whether chromatin accessibility, and therefore gene expression, is affected after an exposure to a sexual signal using epigenomic sequencing techniques. One question we often get is: how do we disentangle the genes that are involved with social interactions and those that are involved in social learning, since social learning is intimately linked with interactions with other individuals? One DEG, the dietary and metabolic glutamate transporter (*dmGlut*), is differentially expressed based on valence in the eyes. In *D. melanogaster*, it is upregulated in isolated flies compared to group-housed flies, implying a role in social interactions (Deanhardt et al. 2023). In our study, since *dmGlut* was differentially expressed in opposite patterns (i.e. modulating valence), and not differentially expressed in the same pattern when exposed to a male (i.e. DE in social situations), this suggests that the expression patterns we see in *dmGlut* are not driven by the presence of another individual, but with the valence of signal associated with the context of learning, therefore disentangling the interactions between social interactions and social learning. These results highlight the molecular mechanisms that regulate the valence of signals and its associated learned behavior, with broader implications for understanding how valence can alter preference or aversion to a visual cue and altering mate preferences.

A number of DEGs identified in both positive and negative mate preference learning contexts have been implicated in classical conditioning pathways, supporting the hypothesis that imprinting learning may utilize similar genetic networks as classical conditioning. There is evidence suggesting that aminergic neurons convey reinforcing signals in classical conditioning in vertebrates and invertebrates (Mizunami et al. 2009), such as dopaminergic and octopaminergic pathways, which have opposing roles in modulating positive and negative learning (Schwaerzel et al. 2003; Mingote et al. 2004; Unoki et al. 2005). Similarly, GABA and glutamate have opposing functions in modulating neural activity, which could aid in modulating positive and negative valence in both classical and imprinting-like learning as well (Siucinska et al. 1999; McCabe et al. 2001; Xiao et al. 2023). This supports the hypothesis that aminergic systems play a crucial role in regulating valence across different forms of learning. Although only a small subset of our DEGs were also found to be involved in classical conditioning in other studies, the presence of such genes supports the hypothesis that at least some components of the genetic architecture underlying classical conditioning may also be recruited during imprinting-like learning, though it may also reflect core genes underlying general learning processes or receptor activity associated with non-learning related processes (e.g. sensory stimuli or handling) after training. For instance, while we did not detect differential expression of immediate early genes such as *c-fos* or ZENK (*Egr-1*), which have been linked to classical conditioning and imprinting in vertebrates (Swank and Bernstein 1994; McCabe and Horn 1994; Bischof 2003; Lieshoff et al. 2004; Mokin and Keifer 2005; Gallo et al. 2018), we did observe *c-jun*, another immediate early gene, downregulated in the antennae during a negative training experience (Fig. 5f). These results suggest that imprinting-like learning and classical conditioning may draw on overlapping neural circuitry to encode for experience-driven learning.

## Conclusion

In this study, we investigated the transcriptomic profiles underlying positive and negative mate preference learning in female *Bicyclus anynana* butterflies. Our results demonstrate that the molecular mechanisms underlying mate preference learning are distinct, tissue-specific, and context-dependent, with sensory tissues showing notable transcriptomic responses that may contribute to how females process and learn cue valence. Furthermore, several genes previously implicated in classical conditioning were differentially expressed in imprinting-like learning, suggesting an overlap in neural circuitry between imprinting-like and classical learning. The identification of shared and valence-associated DEGs, particularly those involved in neurotransmission and epigenetic regulation, suggests potential conserved pathways modulating learning and memory. These findings provide insights into the putative molecular mechanisms driving positive and negative mate preference learning, with broader implications for how these mechanisms can drive preference or aversion for a trait, and facilitate reproductive isolation processes.

## Supplementary Information

Below is the link to the electronic supplementary material.

Supplementary Material 1

Supplementary Material 2

## Data Availability

All raw sequence data associated with this study has been made accessible through the NCBI Sequence Read Archive (SRA) database under BioProject PRJNA1257361. Permutation DEGs’ data and R codes are available at Dryad ([https://doi.org/10.5061/dryad.j9kd51crw](https:/doi.org/10.5061/dryad.j9kd51crw)).
