# Supplementary material for: Divergent sensory transcriptomic profiles in positive and negative learning in Bicyclus Anynana butterflies
Source: J Comp Physiol A Neuroethol Sens Neural Behav Physiol. 2025 Oct 22;212(2):283–96. doi: 10.1007/s00359-025-01771-4 (PMC13086741; doi:10.1007/s00359-025-01771-4)
Supplement: Supplementary file 2 — Supplementary Material 2 [file 359_2025_1771_MOESM2_ESM.docx]

**Article title:** Divergent sensory transcriptomic profiles in positive and negative learning in *Bicyclus anynana*

**Journal name:** Journal of Comparative Physiology A

**Author names:** Yi Ting Ter^1,2^ & Erica L. Westerman^1^

^1^ Department of Biological Sciences, University of Arkansas, Fayetteville, AR 72701, USA

**Email and ORCID of corresponding author:** ewesterm@uark.edu (ORCID: 0000-0002-3575-8298)


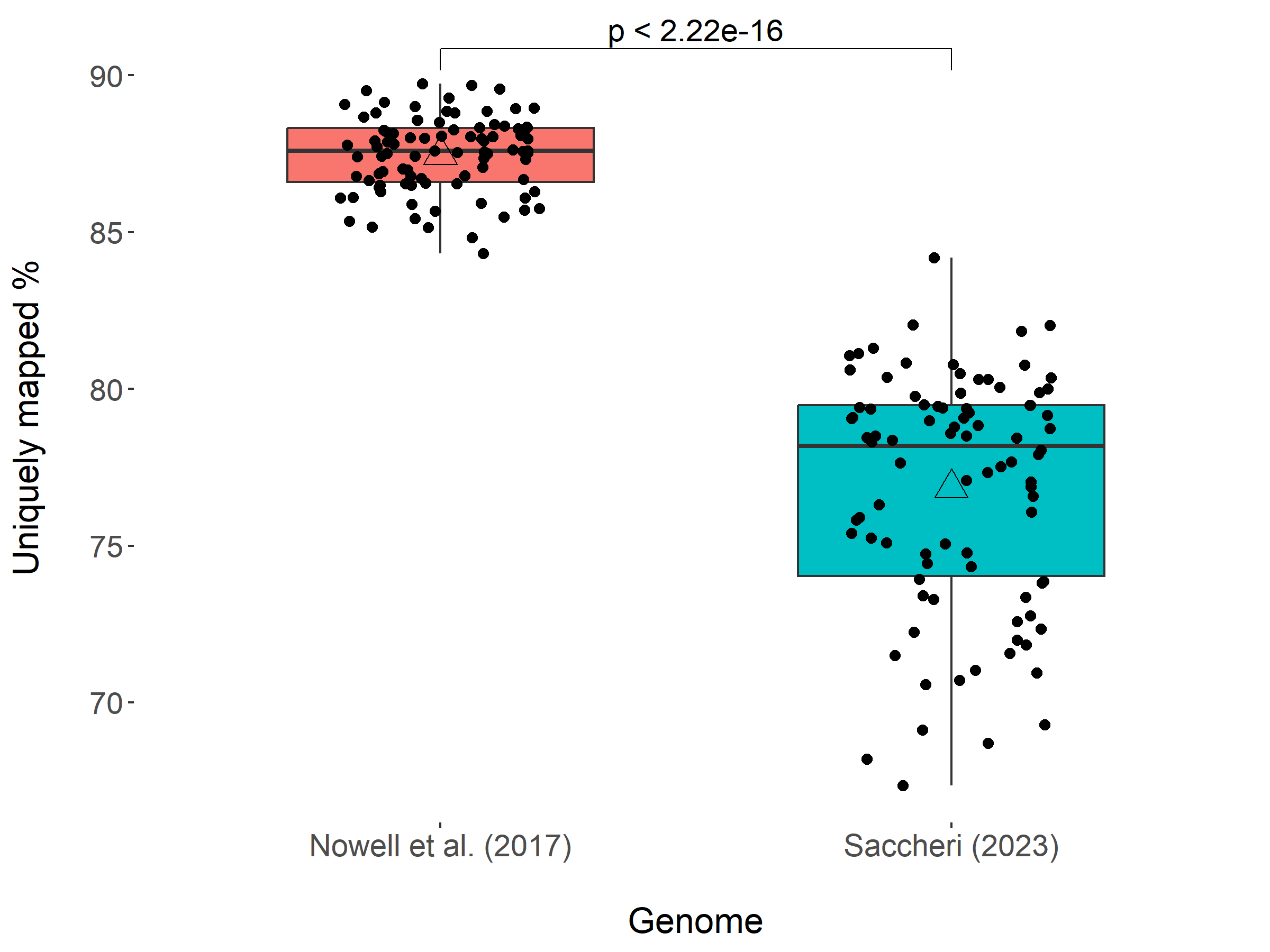

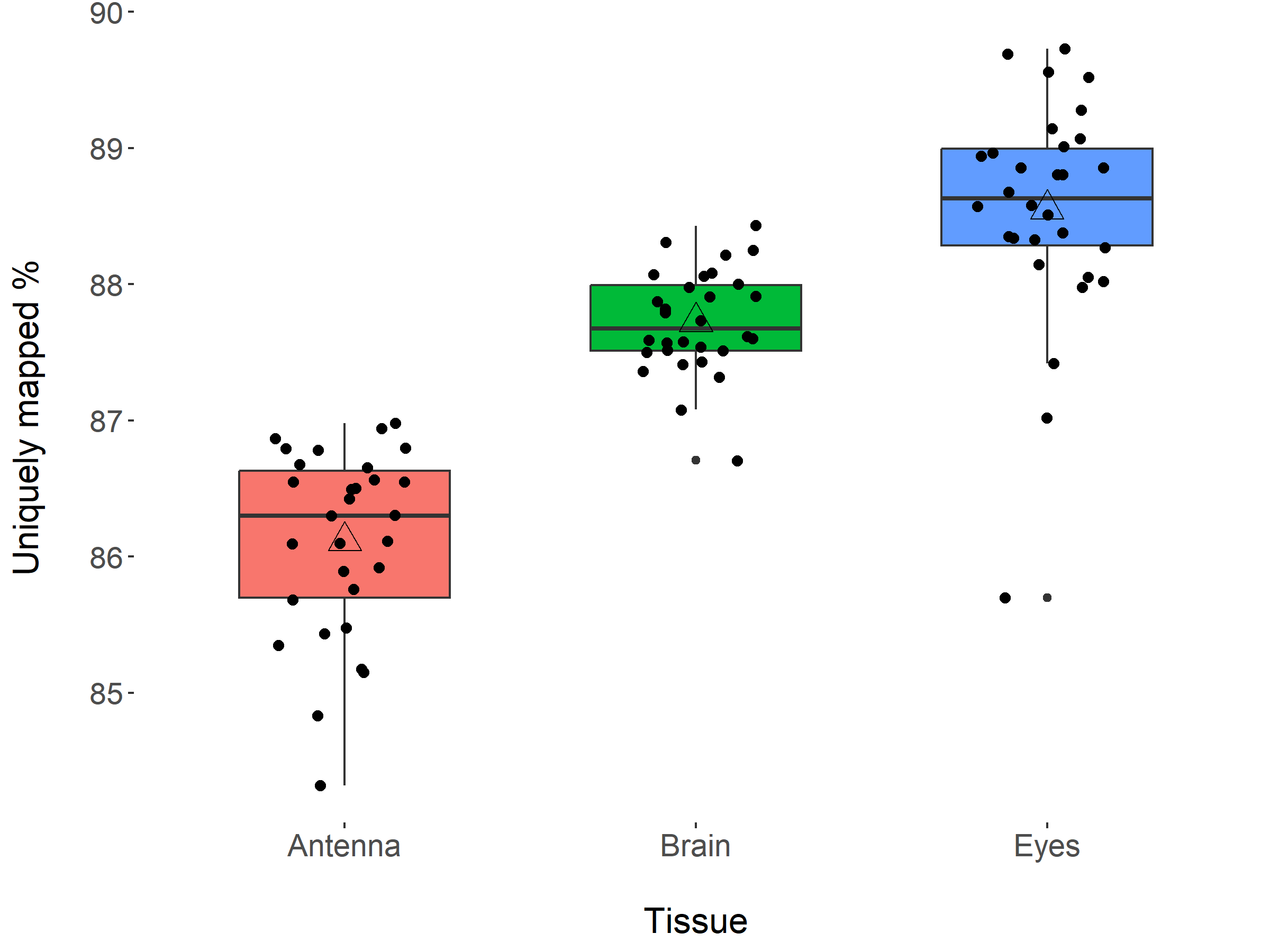


b

a

**Fig. S1: (a)** Uniquely mapped alignment between the previous genome (v1.2), Nowell et al. (2017), and the most recent *B. anynana* reference genome (v1.1), Saccheri (2023). Mean alignment percentage is indicated with a triangle. Our samples had a mean of 87.5% unique alignment to Nowell et al. (2017), compared to a mean of 76.8% unique alignment to Saccheri (2023). **(b)** Uniquely mapped alignment between antennae, brain and eyes using Nowell et al. (2017) genome. Antennae had a mean of 86.1% unique alignment, the brain had a mean of 87.7% unique alignment, and the eyes had a mean of 88.5% unique alignment.


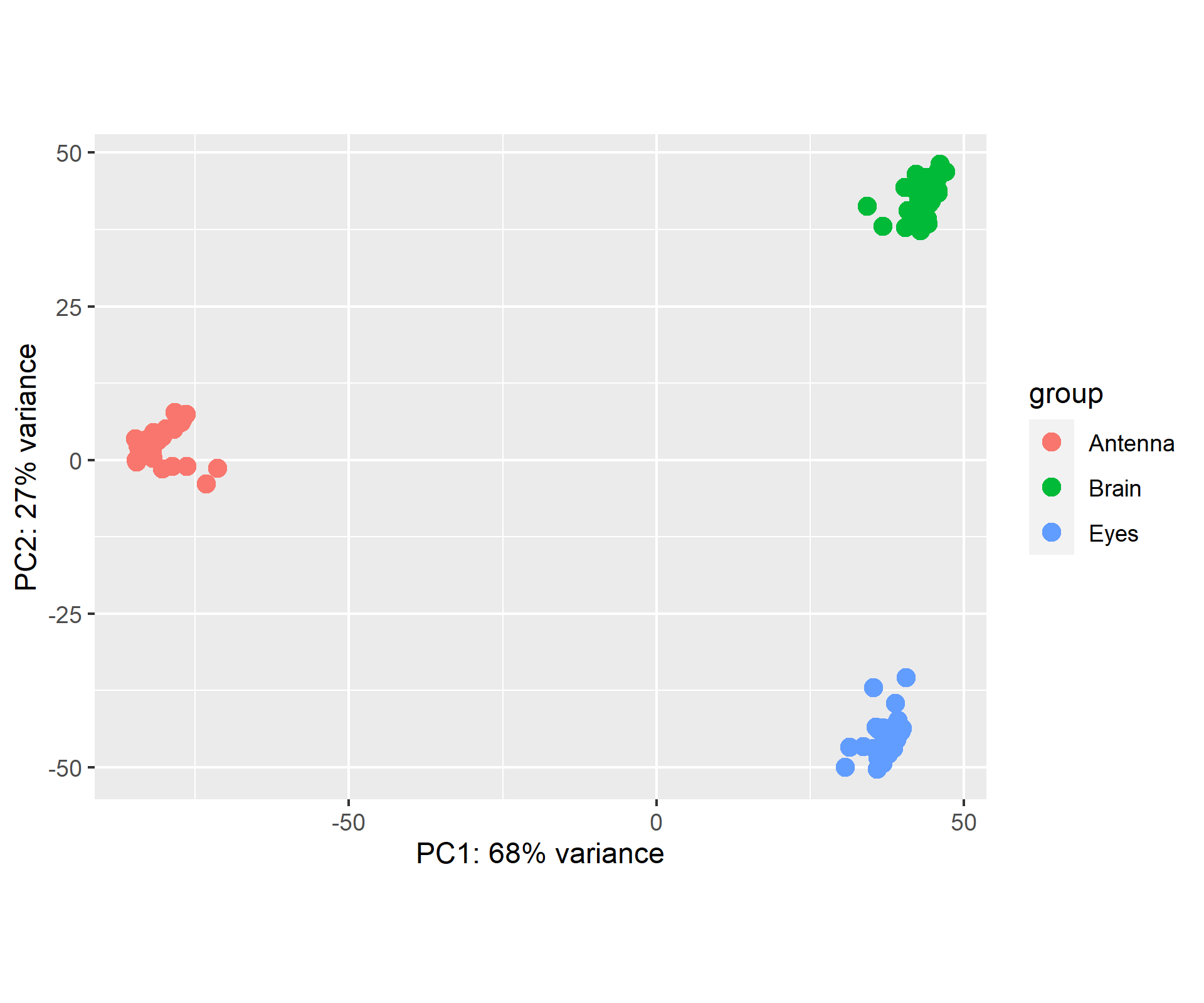


**Fig. S2: Principal component analysis of samples coloured by tissue.** All gene counts have been normalised using variance stabilising transformation before plotting. There was no sample contamination, and the same tissues are clustered together.


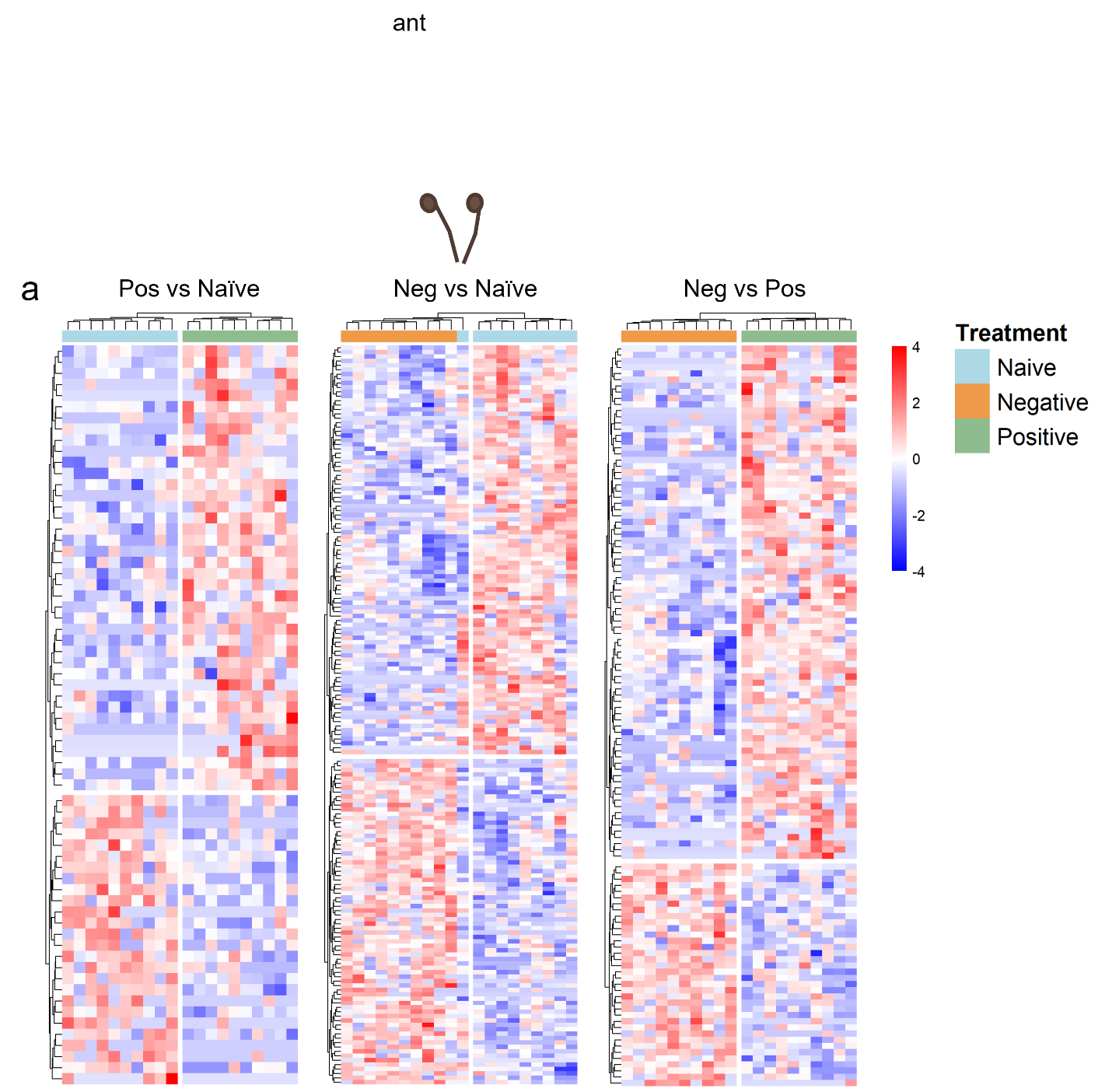


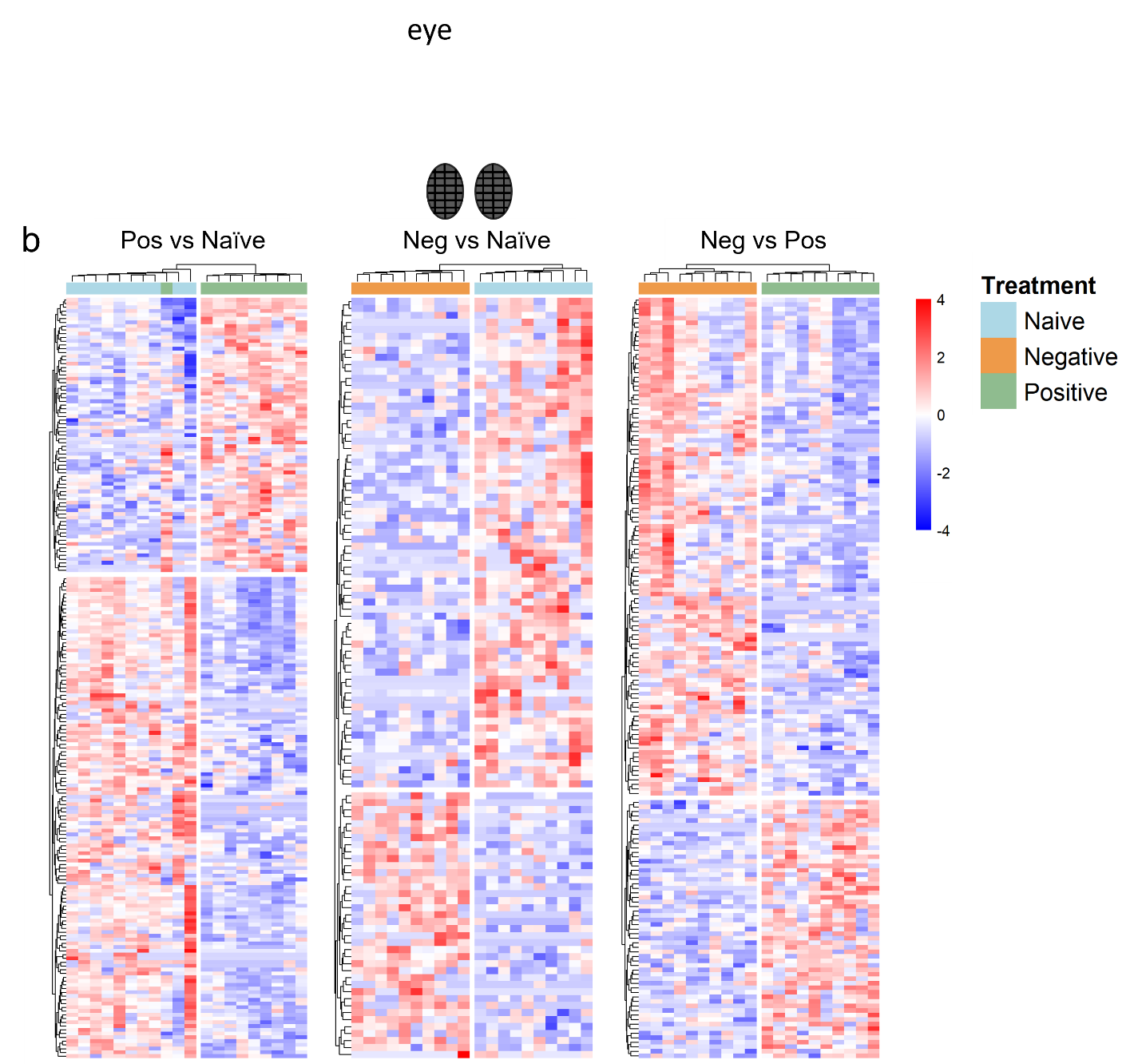

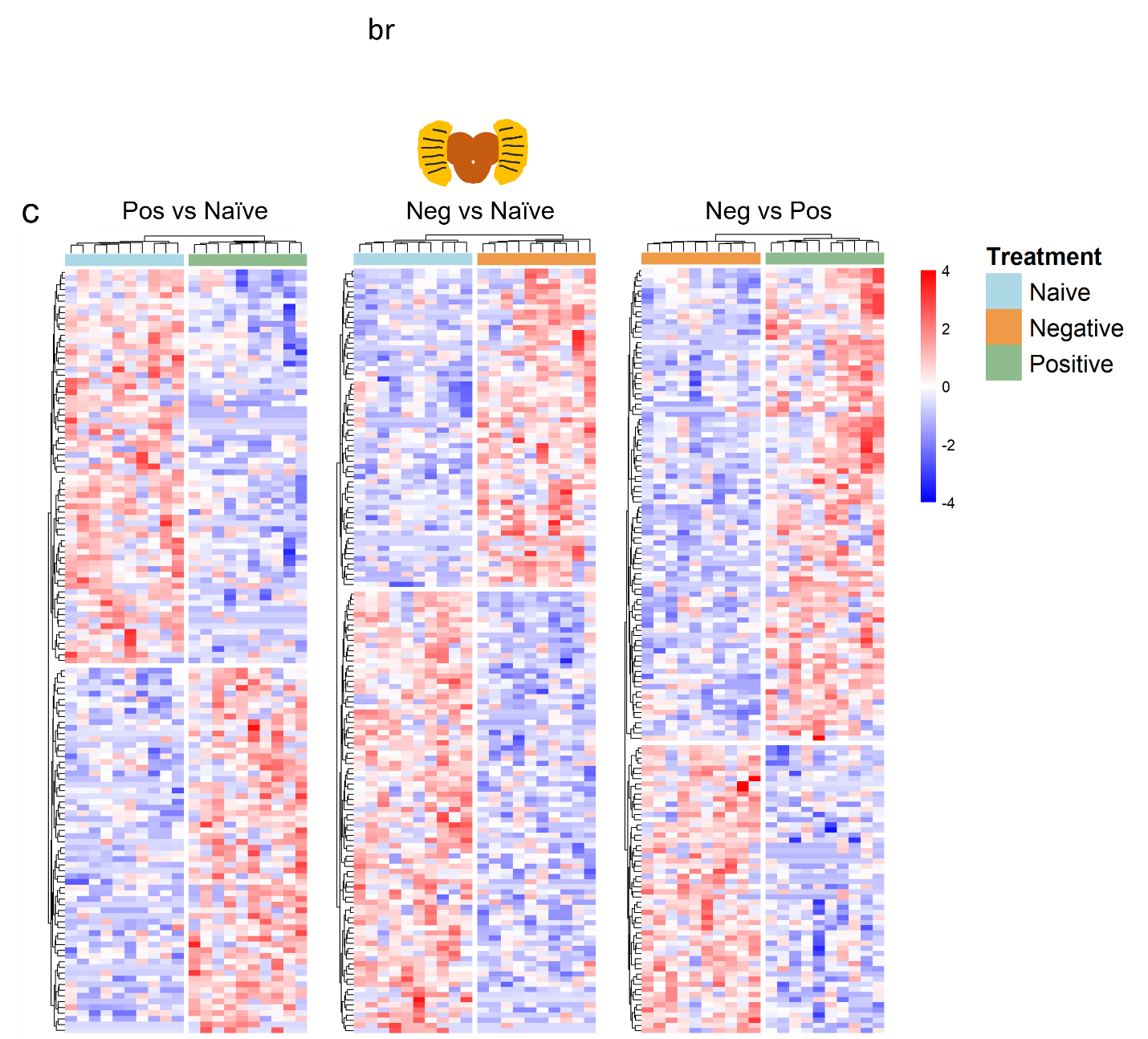


**Fig. S3:** Z-score heatmaps showing differential expression between positively-trained (Pos), negatively-trained (Neg) and naive females across three tissues: antennae (**a**), eyes (**b**) and brain (**c**). Each row represents a differentially expressed gene (DEG), and each column represents a biological replicate. Red colors indicate higher expression, and blue colors indicate lower expression. Treatments cluster together, indicating more differences between than within treatment, except for one sample in the antennae between Neg vs Naïve females, and one sample in the eyes between Pos vs Naïve females.


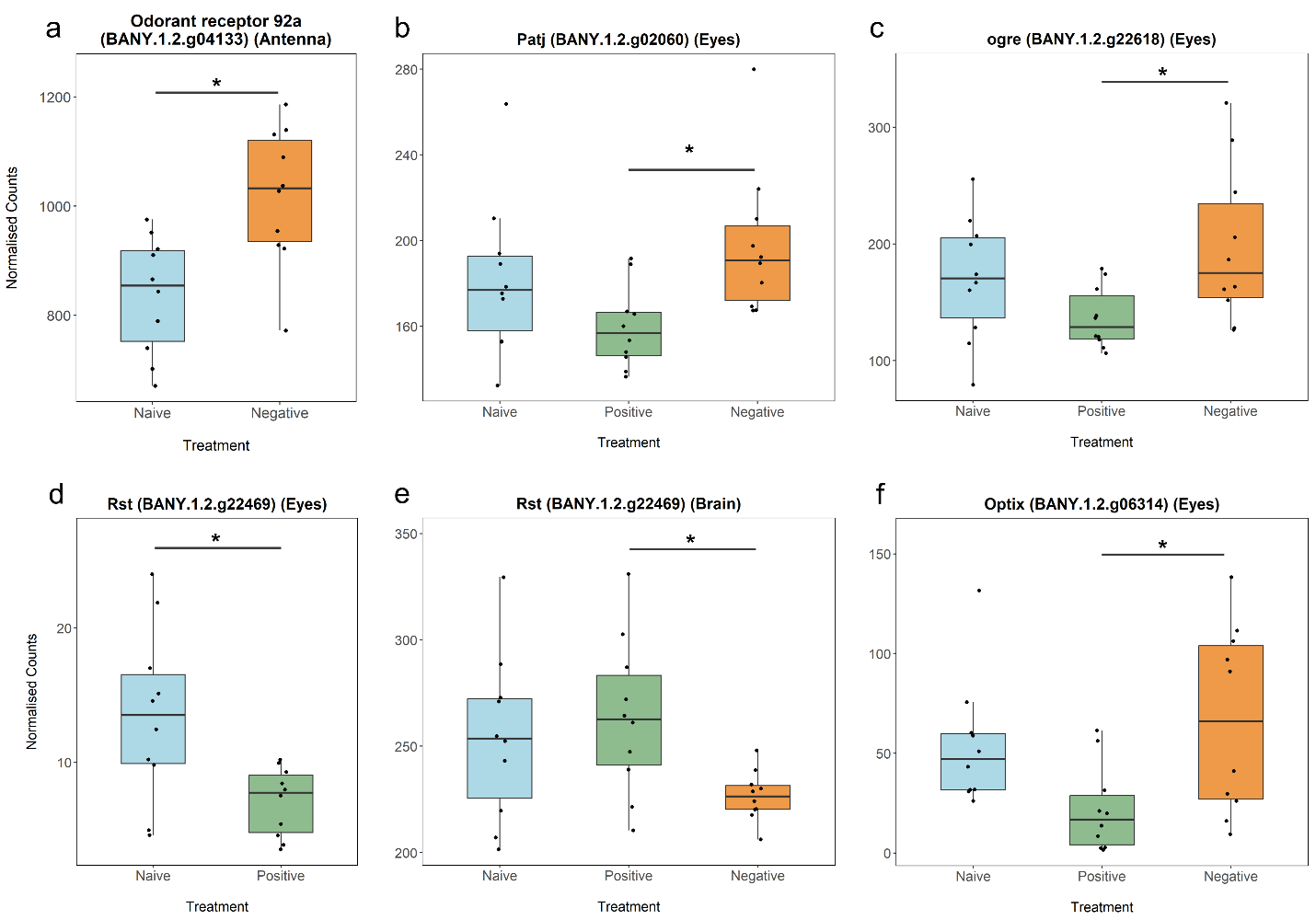


**Fig. S4:** **Box plots of sensory genes.** Differential expression of odorant receptor 92a (*Or92a*) (**a**), *Patj* (**b**), optic ganglion reduced (*ogre*) (**c**), roughest (*rst*) (**d-e**), and *Optix* (***f***) in different valence contexts, across different tissues. Asterisks indicate empirical p < 0.05.

**
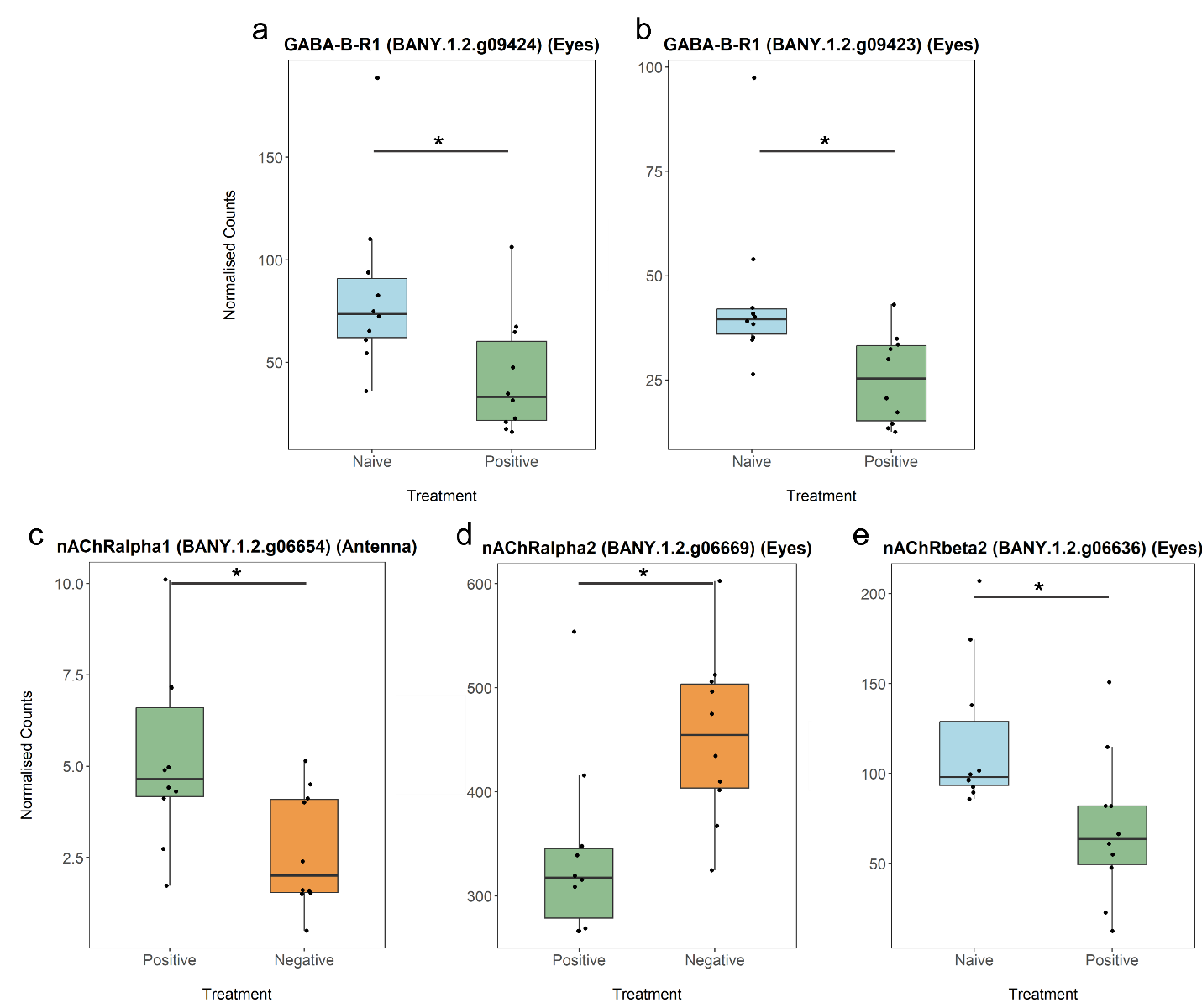
**

**Fig. S5: Box plots of GABA and acetylcholine receptors. (a-b)** Differential expression of *GABA-B-R1* receptors in positive learning, in eyes. **(c-e)** Differential expression of nicotinic acetylcholine receptors (*nAChRs*) in different valence contexts, across antennae and eyes. Asterisks indicate empirical p < 0.05.

**Supplementary Tables**

**Table S1:** Summary statistics for sequenced libraries (± SD). The Mean Mapped Reads column contains the mean number of reads per sample that mapped to the *B. anynana* reference genome (v1.2). All reads statistics are shown in millions.

**Table S2:** DEGs between trained and naïve females in the antenna. Genes are considered differentially expressed in permutation-based DESeq2 analysis when pvalue < perc_1 (1% of p-permutation distribution). The “DE_in” column indicates what valence context (i.e. Positive vs Naïve) the gene is differentially expressed in.

**Table S3:** DEGs between trained and naïve females in the brain. Genes are considered differentially expressed in permutation-based DESeq2 analysis when pvalue < perc_1 (1% of p-permutation distribution). The “DE_in” column indicates what valence context (i.e. Positive vs Naïve) the gene is differentially expressed in.

**Table S4:** DEGs between trained and naïve females in the eyes. Genes are considered differentially expressed in permutation-based DESeq2 analysis when pvalue < perc_1 (1% of p-permutation distribution). The “DE_in” column indicates what valence context (i.e. Positive vs Naïve) the gene is differentially expressed in.

**Table S5:** Putative learning DEGs between trained and naïve females in the antenna. The “expression_pattern” column indicates whether the DEG was upregulated or downregulated in trained females compared to naïve females. DEGs that are similarly expressed (e.g. both upregulated) were defined as putative genes underlying imprinting-like learning, independent of valence.

**Table S6:** Putative learning DEGs between trained and naïve females in the brain. The “expression_pattern” column indicates whether the DEG was upregulated or downregulated in trained females compared to naïve females. DEGs that are similarly expressed (e.g. both upregulated) were defined as putative genes underlying imprinting-like learning, independent of valence.

**Table S7:** Putative learning DEGs between trained and naïve females in the eyes. The “expression_pattern” column indicates whether the DEG was upregulated or downregulated in trained females compared to naïve females. DEGs that are similarly expressed (e.g. both upregulated) were defined as putative genes underlying imprinting-like learning, independent of valence.

**Table S8:** Putative valence-associated DEGs between trained and naïve females in the antenna. These genes were defined as genes having a pattern of high and antagonistic differential gene expression in the positive and negative treatments and intermediate expression in the naïve treatment.

**Table S9:** Putative valence-associated DEGs between trained and naïve females in the brain. These genes were defined as genes having a pattern of high and antagonistic differential gene expression in the positive and negative treatments and intermediate expression in the naïve treatment.

**Table S10:** Putative valence-associated DEGs between trained and naïve females in the eyes. These genes were defined as genes having a pattern of high and antagonistic differential gene expression in the positive and negative treatments and intermediate expression in the naïve treatment.

**Table S11:** No significant differences in activity of naïve females (isolated) and trained females (exposed to a positively valenced male – 4-spotted male with intact sex pheromones) during the exposure time frame (Ernst et al. 2023). Data were non-normally distributed but showed equal variance. W and p-values were obtained using Mann-Whitney tests (n = 10 per group).

| **Female behavior** | **W value** | **p-value** |
| --- | --- | --- |
| Flying count | 106.5 | 0.3606 |
| Flying duration | 121 | 0.628 |
| Flutter count | 86 | 0.1095 |
| Walking count | 89.5 | 0.1189 |
| Walking duration | 119.5 | 0.551 |
| Resting count | 102 | 0.2952 |
| Resting duration | 144 | 0.5451 |
| Antenna wiggle | 113.5 | 0.3584 |
